# Reply to Supady et al. Comment on: Extracorporeal hemoadsorption in critically ill COVID-19 patients on VV ECMO: the CytoSorb therapy in COVID-19 (CTC) registry

**DOI:** 10.1186/s13054-023-04678-1

**Published:** 2023-11-02

**Authors:** J. W. Awori Hayanga

**Affiliations:** Department of Cardiovascular and Thoracic Surgery, West Virginia University, 1 Medical Center Drive, Morgantown, WV 26506 USA

Dear Editor,

We read with interest the letter by Supady et al. [1] and their opinion regarding the methodology and interpretation of the results of the CTC Registry [2]. The content of their critique has, nevertheless, largely already been covered within the limitations cited in the original publication. These included but were not restricted, to the absence of a control arm, potential for selection bias, limited sampling of biomarkers and the inability to determine whether the high survival rates were attributed to CytoSorb, ECMO or indeed both. We wish to point out that all patients were treated according to the FDA’s Emergency Use Authorization-EUA) and we disavow them of the notion that the determination of early vs. late treatment subgroups was an “arbitrary” exercise. Finally, Supady et al. conjecture our position as “unsubstantiated claims” with an ill-advised position that CytoSorb should only be used in the setting of controlled clinical trials.

The methodology and results of the CTC Registry as discussed in the original publication require little further clarification as written. Nevertheless, we remind Supady et al. that the CTC Registry is the largest multicenter experience with the use of CytoSorb in patients on ECMO with severe COVID-19 respiratory failure. An additional strength of the CTC Registry was that patient selection and treatment parameters were each guided by predefined EUA criteria,  thereby providing standardization across participating sites in a manner emblematic of prospective controlled studies. Furthermore, the safety profile of CytoSorb in the CTC registry was evaluated under FDA Investigational Device Exemption (IDE) regulations and monitored according to EUA standards.

Importantly, the main finding of the CTC registry is that survival with the combined use of CytoSorb and ECMO in appropriately selected patients compared favorably to the global experience of ECMO use in COVID-19 as reported by the ELSO Registry. That observation coupled with the fact that no device-related adverse events were reported by any of the participating sites directly silences any concerns about the safety and effectively refutes the notion CytoSorb should be limited to controlled clinical trial settings. In fact, the external validity of the multicenter CTC registry incrementally advances the evidence base for the use of CytoSorb with ECMO in critically COVID-19 patients that has been previously limited to single center reports, much like Supady’s own experience [3].

Finally, the proposed concept of “enhanced lung rest” via a combination of ECMO to rest the lungs and hemoadsorption to address concomitant hyperinflammation is a novel and intriguing therapeutic strategy that is based on sound pathophysiologic considerations. However, what is not novel, is the observation that early intervention improves outcomes as this has become established approach in critical care medicine. In aggregate, the CTC results are encouraging and support the rationale for further investigation of early initiation of enhanced lung rest in controlled clinical studies.

## Data Availability

Not applicable.
